# Documenting, Protecting and Managing Endangered Maritime Cultural Heritage in the Middle East and North Africa (MENA) Region

**DOI:** 10.1007/s11457-022-09338-z

**Published:** 2022-09-20

**Authors:** Colin Breen, Lucy Blue, Georgia M. Andreou, Crystal El Safadi, Harmen O. Huigens, Julia Nikolaus, Rodrigo Ortiz-Vazquez, Nick Ray, Ash Smith, Sophie Tews, Kieran Westley

**Affiliations:** 1 School of Geography and Environmental Sciences, Ulster University, Northern Ireland, UK; 2 Archaeology, School of Humanities, Southampton University, England, UK

**Keywords:** Maritime cultural heritage, MENA, Climate change, Development

## Abstract

For millennia, the Middle East and North Africa (MENA) region has been a culturally dynamic zone, bounded by maritime societies dependent on the sea for communication, trade and livelihoods. The archaeological evidence of these past societies represents an extraordinary physical legacy of human endeavour and presence across this region, contributing to senses of place, identity and belonging amongst contemporary coastal communities. However, the coastal landscapes and marine environment of the MENA region are undergoing a period of profound change, associated with large-scale human development and climate change. In order to assess this change and the level of impact on the resource, the Maritime Endangered Archaeology project (MarEA) was established in 2019 to document cultural heritage sites and landscapes across the coastal and near-shore zones of the survey region. This paper introduces the work of the project and outlines a series of case studies presented in this volume that are representative of the variety and depth of work being undertaken within the project.

## Introduction

For millennia, the *Middle East and North Africa (*MENA) region has been a culturally dynamic zone, bounded by maritime societies dependent on the sea for communication, trade and livelihoods (Broodbank 2013; Bewley et al. 2016; Andreou et al. 2020). From the earliest fisher communities through to Bronze Age Phoenician traders and the empires of Greece and Rome, the communities who lived across this region both depended upon and were intrinsically connected to the sea. Subsequently, kingdoms and polities continued to vie for control of these coasts and seaways, while their strategic and economic importance continued into modern times. Thousands of years of human activity have left significant archaeological remains, ranging from coastal castles, port structures and shipwrecks to structures like fish traps, coastal quarries and landing places. This evidence of past society represents an extraordinary physical legacy of human endeavour and presence across this region, and the region’s archaeology contributes to senses of place, identity and belonging amongst contemporary coastal communities. These sites and landscapes represent a major facet of the tangible marine cultural heritage (MCH) of the region, while also generating many elements of intangible heritage through cultural traditions including folklore, stories, songs and belief systems. Yet, this resource is facing unprecedented levels of threat and impact from both natural and human processes of change. The Maritime Endangered Archaeology project (MarEA), funded by the Arcadia Fund, was established in 2019 to document and quantify this threat, and provide a baseline assessment of the nature and stability of these sites and landscapes (Andreou et al. 2020). This volume presents some initial results from the project, through the publication of a series of important area and regional case studies. These are intended to highlight the nature and extent of threat impacting these sites and landscapes, and provide a historical and cultural context for these places. It is envisaged that these studies will be used to further inform the continuing development of management and protection strategies around these significant sites, and work towards their future protection in the face of growing threats.

### Marine Cultural Heritage Impacts and Threats

The coastal landscapes and marine environment of the MENA region are undergoing a period of profound change (Breen et al. 2021; Westley et al. 2021). As the countries of the region continue to experience conflict and economic instability, a number of trends are apparent. A marked increase in rural–urban migration to the coastal towns and particularly cities, as a direct consequence of deteriorating rural livelihoods (Waha et al. 2017), has placed considerable stress on access to housing, inevitably leading to large-scale building and redevelopment. Much of the coastline has been subject to a period of hyperdevelopment as coastal industry and infrastructure are expanded. This, in turn, is leading to ever-increasing competition for development space along the coast. Historic port cities are particularly susceptible to these pressures, with their historic cores and waterfronts deemed especially attractive for redevelopment. While the adjacent coastlines may have a less visible heritage value, they also contain significant elements of past maritime activity, and warrant equal levels of attention in terms of assessment and protection.

While the drivers of economic and development change are radically altering historic coastal landscapes, natural drivers of change are also impacting upon the resource. Across the region, extreme weather events are directly affecting livelihoods and household income, with agricultural and fishing practices coming under particular pressure, leading to migration and displacement (Wodon et al. 2014). For instance, both mean and extreme temperatures have increased in recent years and are projected to increase further in the coming years in North Africa and the Arabian Peninsula. Precipitation is also projected to change, but with regional variation. North Africa will probably suffer from increased aridity, but the Arabian Peninsula could experience increased frequency and intensity of extreme rainfall events (IPCC 2021). These changes are particularly significant given that 75% of agricultural practice across the region is rain-fed, and thus highly exposed to changes in the precipitation pattern or changes in heat (Waha et al 2017). All coastal areas in MENA will experience sea-level rise of 0.28- 1.02 m by the year 2100 resulting in increased coastal flooding and erosion (IPCC 2021). It is estimated that 75% of all building and infrastructure across the region are at risk from the impact of climate change, and from sea-level rise and storm surges, particularly prevalent along the coast (Göll 2017). Historic buildings and structures, both at and near the coast, are again particularly susceptible as many tend to have been built close to the water’s edge, and both their age and structural character make them more prone to deterioration. The combination then of both natural and anthropogenic threats, in a region marked by seemingly endless geopolitical contestation, coupled with weak environmental and heritage resource governance and limited capacity, makes the documentation and protection of the resource an urgent priority.

### Maritime Endangered Archaeology Project (MarEA)

In an attempt to quantify the impact of these processes on the heritage resource, the Endangered Archaeology in the Middle East and North Africa (EAMENA) project has documented an increasing level of threat acting upon the terrestrial archaeological resource across the region, ranging from conflict, development, agricultural activity and climate change (Bewley et al. 2016; Sheldrick and Zerbini 2017; Rayne et al. 2017). However, this Oxford University-centred project focusses primarily on terrestrial hinterlands. Through a working partnership with EAMENA, the MarEA project was established in 2019 to document the coastal zones of the survey region in a comprehensive manner, noting all coastal, near-shore and underwater sites visible from satellite images and recorded in extant documentation. It mirrors the EAMENA regional scope, but aims to provide a quantitative assessment of nature and, where possible, condition of the MCH resource, as well as any potential threat faced.

## Methodology

**Fig. 1:**
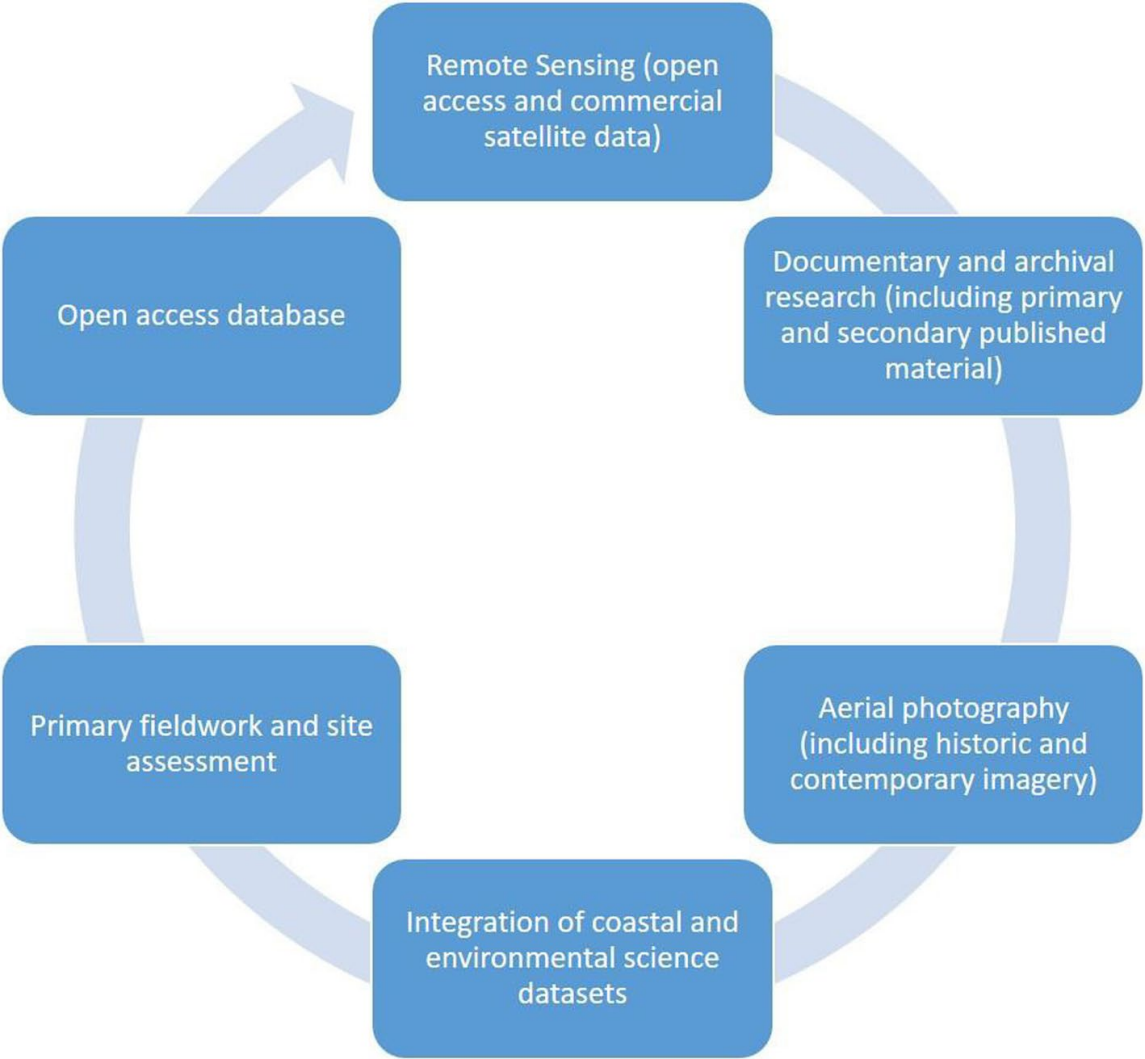
Methodological cycle of the MarEA project

The MarEA project aims to document the MCH of the entire coastal and near-shore zone both on land and underwater across the MENA region (Andreou et al. 2020). Through a partnership of the Universities of Southampton and Ulster, the MarEA team have adopted the principles of the EAMENA project with respect to the methodology of documentation, data capture, storage and dissemination (Fig. 1). Documentation is discussed in more detail below while data capture, storage and dissemination are enabled by the EAMENA open-access database. This graph-based database was developed by EAMENA using the Arches platform and is hosted at University of Oxford. It captures a range of information on archaeological sites including basic data such as site location, function, interpretation and period, but also records information on past disturbances affecting the site, potential threats and the overall site condition. Consistency and accuracy are maintained by the use of fixed resource models and controlled vocabularies. Although initially developed for EAMENA’s (primarily terrestrial) purposes, collaboration between MarEA and EAMENA has extended the database through development of terminologies and information categories specific to maritime and coastal archaeological sites, and a new geoarchaeological resource model which aims to capture evidence of coastal environmental change has been added. These extensions facilitate inclusion of all maritime archaeological sites (encompassing coastal as well as near shore/underwater, including reference to deep water (see below) sites). The database is freely available to bona fide users but also has built-in mechanisms to restrict access to documented sites in the case of improper use.

In order to determine the nature and degree of threat to the MCH, the MarEA team documents the changing nature of the character and context of maritime archaeological sites. An appreciation of local to regional-scale sea-level change, sedimentary and erosional processes, and other drivers of change, provides a framework within which to identify and interpret coastal and underwater sites with respect to their location both past and present. The relationship of maritime sites to the sea is often dynamic and changeable over time and space, owing to the aforementioned processes. Understanding coastal change is then central to understanding the form and function of archaeological features and their changing relationship to coastline and the sea over time (Fitzpatrick et al. 2015). By integrating archaeological and marine science, the project will create an enhanced documentation record and be positioned to make an assessment of particular threats to these endangered maritime sites.

### Documentation

The MarEA documentation methodology, initially informed by the Honor Frost Foundation, funded the *Syria Benchmarking Project* (HFF 2019; https://honorfrostfoundation.org/2020/06/25/tyre-lebanon/)*.* This 1-year project mapped, using satellite imagery and extant published data, the archaeological sites and features, both coastal and underwater, along the length of the Syrian coast. Additionally, this project was conducted in liaison with the EAMENA team and adopted both its methodology and data management/database systems. A developed MarEA documentation strategy was then strongly informed by this experience, which included modifications to the EAMENA database (see above). It centres on the collation and interpretation of satellite imagery (mainly open-access Google Earth and declassified Corona spy satellite images, supplemented where required by imagery from other sources, including commercial), together with an analysis of all published data and archival images (e.g. historic aerial photos and maps) relating to the coastal and near-shore archaeology of the MENA region. Additionally, where available, grey literature and data (e.g. offshore industry and coastal developers, local governments, unpublished theses) are also sourced and included within the interpretation framework.

The analysis process is systematic and can be seen as an example of the ‘brute force’ approach to archaeological interpretation of remotely sensed data (Casana 2014), enabling large areas of landscape to be rapidly assessed and recorded using satellite data. Similar to the EAMENA strategy, MarEA has divided the total survey region in 20 × 20 km grid squares. Each square is examined systematically and, in turn, using all the available aforementioned data sources. The ultimate aim is to create records and document for each individual square site. While the core documentation work is based on remotely sensed imagery, field survey and ground checking (where feasible) also feed into this process (see Field Documentation). Importantly, given the focus on endangered archaeology, these records can include detailed assessments of past disturbance (i.e. adverse impacts; recorded data include causes, effects and dates of disturbance, potential threats (i.e. likely future impacts; recorded data include causes and likelihood of threat) and site condition). In all cases, uncertainties or nuances can also be accounted for through use of accompanying categories, such as ‘Possible/Probable’ for future likelihoods and ‘High/Medium/Low’ for past impacts. Nevertheless, it should be stressed that documentation is comprehensive as well as systematic; the aim is to capture all sites regardless of whether they have been disturbed or are at risk. After all, a site that has not been disturbed in the past might still be prone for disturbance in the future. Comprehensive documentation therefore enables an active record which can be updated as required.

The process of identifying, collating, documenting and interpreting sites in both the coastal and near-shore zones is a complex procedure due to the nature of the resource. In these environments, archaeological sites are subject to a variety of ever-changing physical and anthropogenic processes that can result in rapid site degradation and dispersal. The dynamic changing environment associated with past and present climate and environmental change, coupled with extensive urban expansion and maritime development, often makes identification and management problematic. This is further compounded by the sheer density of sites in this region. The challenges of identifying sites underwater also present further issues particularly because satellite imagery only gives a clear picture of the seabed in shallow, clear waters. Although advances have been made using recent developments in air and space-borne remote sensing of the seabed for archaeological purposes, such as Satellite-Derived Bathymetry (SDB) (Guzinski et al. 2016), LiDAR/laser bathymetry (Doneus et al. 2013) and hyperspectral imaging (Guyot et al. 2019), these are either not available for much of the MENA region or, in the case of widely available SDB, more useful for landform detection and palaeo-geographic reconstruction than archaeological site prospection (Guzinski et al. 2016; Westley 2021). More reliable methods for identifying archaeological sites in deeper or turbid waters using ship-borne marine geophysical techniques are well established (Menna et al. 2018). However, while the resulting data (e.g. high resolution bathymetry) are becoming widely available in parts of the world, thus enabling usage for archaeological purposes (Plets et al. 2011; Westley et al. 2011; Davis et al. 2020; Majcher et al. 2021), the same is not yet true of the study area. Either the requisite surveys have not been conducted or data have been collected, but are not publicized or readily accessible for commercial, military or governmental reasons, or simply because open-access provisions are lacking. Redressing this will involve the identification, analysis and integration of existing deeper water datasets into the MarEA platform, and will also involve engagement with commercial marine exploration companies and hydrographic surveyors in an effort to identify and consider the marine archaeological potential of their datasets. This will form an integral part of the network-building activities central to this project. Such approaches are also aimed at building relationships with the marine industry and other data producers, to access data and encourage better, more informed practice relating to the marine heritage resource. The building of relationships with big data collators should prove to be a long-term benefit to the MarEA project and an appreciation of regional context. As of yet, the project has not developed a structured way of undertaking this form of industry data acquisition, and it remains on an ad hoc basis. One strategy being developed is through the development of government protocols that allow for data sharing across departments.

An initial phase of rapid assessment of resources took place over the first two years of the project in order to achieve a comprehensive geographic coverage and develop preliminary understandings of the nature of the resource and any associated threat levels. Sites of particular importance or those prone to high levels of threat are given a higher degree of analysis. The second phase of the project (years 3–4) will target specific landscapes and sites that will be subject to more intensive analysis and documentation, based upon an analysis of threat or potential, while ongoing wider geographic documentation will also continue.

### Field Documentation

Field survey and documentation are also an intrinsic part of the project. There are currently two strands to this. Strand A involves field survey to enhance the documentation of coastal and near-shore underwater sites. This includes targeted coastal and marine survey and ground checking survey areas, through prioritizing areas of imminent threat or high archaeological potential. Currently, the preferred delivery mode for this strand is to engage with in-country teams and support the development of maritime archaeological capacity within these teams. These can be drawn from government, university, NGO or community partners, and further enhances the partnership approach being adopted by the project. The partnerships arose directly through the MarEA project and developed through a combination of existing networks and connections, as well as through a concerted effort to support regional capacity development. The realization of such a network faced considerable challenges in terms of limited national funding in many countries, coupled with limited in-country expertise and an associated peripheral awareness of the marine cultural heritage resource. Ongoing regional training programmes and the direct provision of funding support to in-country partners have helped overcome some of these issues, but conflict, regional instability and limited political interest continue to pose significant barriers to the longer-term sustainability of this work. To date, a number of these surveys have been conducted in Libya (Nikolaus et al. this volume), but due to the recent COVID-19 pandemic, other planned field interventions such as in Egypt and Bahrain have been postponed. The second Strand B involves emergency reactive documentation. The threat level the maritime cultural resource is subject to has already been discussed, and the MarEA project is acutely aware of both the impact this is having on site stability and protection and the often limited local capacity that exists in some countries to be able to respond adequately to these impacts (Demesticha 2019). Thus, built into our practice is the ability to respond to particular circumstances or events and undertake emergency documentation using a variety of maritime archaeological methodologies and techniques. The project team is able to respond to particular appeals for assistance and collaboration from in-country partners or network associates to provide professional advice on the documentation and protection of sites under threat. This might occur in advance of major infrastructural development, natural disaster or from conflict. It will also further facilitate elements of capacity building with regional colleagues. This approach is currently being adopted in Egypt, Algeria and Bahrain. Finally, a series of more intensive, underwater documentation and verification projects will also be undertaken across the time frame of the overall project. This will involve site-specific diving and marine geophysical surveys of underwater sites and detailed underwater documentation at particular places. A number of factors will be involved in site selection including potential, threat, access and safety, but will be primarily guided by in-country needs and to support capacity development.

### Future Protection and Management of Resources

**Fig. 2:**
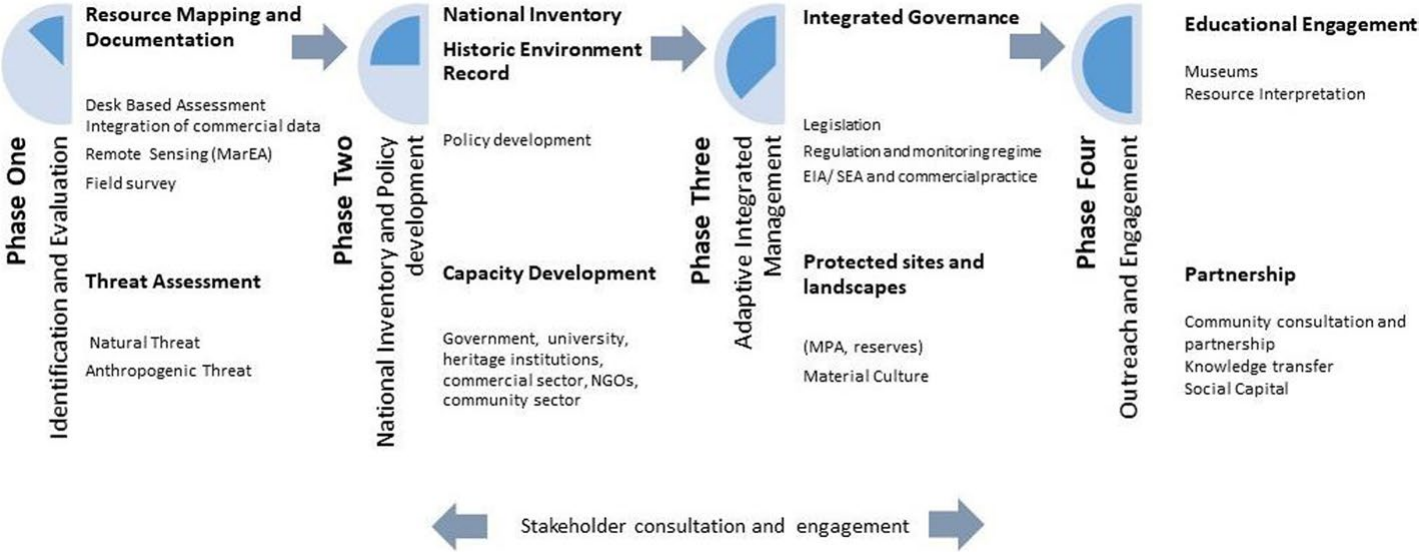
A conceptual developmental model for how MCH management and capacity could develop in the coming years

The project team is conscious that documentation and site recording are only one facet of a larger process that is required to ensure resource protection and sustainable management. The current project is focussed on phase one in the linear progression model, detailed below (Fig. 2). With any marine resource management strategy, the crucial first step is a quantification exercise that creates a baseline assessment of the nature, extent and condition of the resource. An integral part of these is the development of a full threat assessment from both natural and anthropogenic sources.

Such a baseline assessment could then form the basis of national inventories of sites, monuments and historic landscapes. While many countries across the region have developed such records, data relating to the coastal and underwater environment are often limited or absent. Currently, the maritime cultural resource is little understood across all of these regions. Governments have little input into its protection and management, and there has only been limited research conducted into the resource, most of which has been undertaken by researchers from outside the region. The broader impact of this project therefore is to provide, through working in partnership with in-country partners, management tools to the governments in the region, through significantly enhancing an understanding of the nature and extent of the resource, and the range and character of the threats the resource is exposed to. This will enable regional managers to be able to identify the key priorities for site management and facilitate the future management of the coastal and underwater resource. MarEA will work with regional authorities to support the development of in-country capacity to fully accommodate these data and work towards marine-inclusive historic environment records.

The documentation and recording of these sites and places are only one part of a much broader strategy towards the realization of a framework of integrated governance focussed on the protection of marine environments and the cultural heritage resource. This will involve legislative protection and the accommodation of MCH in the regulatory and monitoring frameworks of a country’s approach to environmental and resource management. Full consideration of MCH with commercial development practice will also need to be realized, specifically within the Environmental Impact Assessment (EIA) or the Strategic Environmental Assessment (SEA) structures. This is particularly critical to the archaeology of the coastal and near-shore underwater zone, which is frequently overlooked by ministries responsible, due to lack of knowledge and in-house expertise. Therefore, this project aims to redress this imbalance, encourage specific government engagement with the resource and generate capacity in the region (Recinos and Blue 2019). This will involve the development of regional and national policy that will enhance resource protection, and encourage an improved legislative framework for resource management. Through parallel programmes of capacity building outside the Arcadia funding framework, we aim to develop the existing limited capacity base and develop a professional network of maritime archaeologists and heritage managers. In terms of further impact, this project will significantly enhance the visibility of the maritime cultural resource amongst the broader research community, as well as amongst the coastal communities that live and work across these regions. These documentation data will also be available to researchers from the multiple disciplines that work in the marine environment, and will be available during the decision-making process for marine development and coastal protection projects.

Closely aligned to this stage is ensuring adequate management measures are in place to establish the continued protection and controlled access to the resource. This may range from individual site protection measures to area reserves and adequate conservation facilities. Throughout each of these stages, an intensive and informed programme of engagement with all marine and heritage users is imperative. This is both a top-down and bottom-up process, where all users have equitable access to the data and decision-making process. A detailed programme of engagement with wider archaeological networks and projects will also be developed throughout the various stages of the overall project. This will include focussed engagement visits across the target countries to promote and engage partners and other data providers with the project, as well as further work to engage with projects from other disciplines to quantify the nature of their data and its accessibility. Through the development of deeper knowledge of both natural and cultural resources of marine protected areas, and how these have changed over time, an integrated approach is likely to be a critical factor in providing effective stewardship of these significant sites (Barr 2013). Such an approach acknowledges the complexity of the resource and will facilitate more effective management (Vrana and Vander Stroep 2003).

### Case St﻿udies

The papers in this volume present a series of important case studies of the current work of the MarEA project. They represent a baseline assessment of the nature and current condition of the MCH resource across the MENA region and provide a detailed analysis of the range of threats impacting this resource. Coastal environments are dynamic entities, subject to a series of both natural and cultural drivers of physical change. In Syria, baseline assessment has documented both contemporary and historical change along the coastline (Westley et al. this volume). The combined natural processes of eustatic sea-level change, tectonic movements, erosion and sedimentation have resulted in a continually changing coastline and led to the associated adaptation of settlement patterns and human activity by past societies. Coastal change will continue into the future, driven heavily by climate change-induced sea-level rise and surges. This threatens Syria’s under-researched but invaluable MCH and adds to the considerable anthropogenic pressures (e.g. construction, infrastructure development) experienced by this coastline.

One of the core strategies of the MarEA project has been to develop area-specific projects that involve an intensive investigation of the natural and cultural environments of these locations. In a joint MarEA/EAMENA project, researchers have been investigating the threats and disturbances to cultural heritage in South Sinai. In particular, the study is concerned with a comparative analysis between the coastal areas and the world heritage area of St. Catharine’s Monastery (Tews et al. this volume). Using remote sensing as its core methodology, this project assesses the extent of threat impacting upon the resource and forwards a number of recommendations on how the resource can be protected and managed into the future. On the Egyptian Mediterranean coast, particular geographic emphasis has been placed on the north west coastal region, in particular the modern city and ancient port site of Marsa Matruh (Ray et al. this volume). Here, research has focussed on the impact of modern port expansion and urbanization on the cultural heritage resource and how the coastal landscape has been subject to continual change from natural, but primarily, anthropogenic processes. Remote sensing and documentary research have facilitated deeper understandings of the historical development of human activity in this area.

A related case study has focussed on the Cyrenaica coast of Libya. MarEA, in partnership with local government and university archaeologists, has been involved in a coastal survey of this highly dynamic littoral. A centre for human activity for many thousands of years, this coastline contains impressive remains of past societies. However, it is a coast subject to significant pressures from erosion, storm impacts and development. This paper reports on the results of both the remote sensing and survey work undertaken by Libyan archaeologists and addresses the future management needs in this volatile political environment (Nikolaus et al. this volume). It also highlights the strategic partnership approach MarEA is endeavouring to implement across the region. Further west, in Morocco, a team of archaeologists have been working for a number of years mapping maritime cultural landscapes on both the country’s Mediterranean and Atlantic coasts. This paper presents a broad introduction to the nature and chronology of the MCH resource of Morocco and documents the threats facing the resource. A number of future research strategies are also presented (Trakadas et al. this volume).

The impact of natural events on MCH is of pressing interest to MarEA. On the southern Oman coast, research has focussed on the impacts of cyclones on MCH. This paper contextualizes cyclone events affecting the Arabian Peninsula, with a specific focus on Oman and the Dhofar Governorate (Andreou et al. this volume). An analysis of the impacts and a series of observations are presented from locations like the Islamic site of Al Baleed. As with other papers, a series of future research strategies are also presented.

## Conclusion

The MarEA project represents a significant development for the future protection and management of marine cultural heritage across the MENA region. For the first time, the resource is being systematically mapped and documented on a regional basis. These data, available through the EAMENA open-access online database, will provide an important baseline assessment of the nature and condition of the resource and identify the threats and impacts the resource is subject to. The scale and impact from both natural and anthropogenic sources are alarming, and regional governments and relevant authorities need to urgently address the rapid loss of sites and monuments. But documentation is just one of a number of important steps that need to be taken. It must take place in tandem with a programme of capacity development, community engagement and field investigations. The partnership approach being adopted by the project will contribute towards this process, and MarEA will continue to work with our regional partners to realize better understandings and protection for the marine cultural heritage resource.
